# The 'Healthy Dads, Healthy Kids' community effectiveness trial: study protocol of a community-based healthy lifestyle program for fathers and their children

**DOI:** 10.1186/1471-2458-11-876

**Published:** 2011-11-19

**Authors:** Philip J Morgan, David R Lubans, Ronald C Plotnikoff, Robin Callister, Tracy Burrows, Richard Fletcher, Anthony D Okely, Myles D Young, Andrew Miller, Victoria Clay, Adam Lloyd, Clare E Collins

**Affiliations:** 1 Priority Research Centre in Physical Activity and Nutrition, University of Newcastle, Callaghan, NSW, Australia; 2 School of Education, Faculty of Education & Arts, University of Newcastle, Callaghan, NSW, Australia; 3 School of Biomedical Sciences and Pharmacy, Faculty of Health, University of Newcastle, Callaghan, NSW, Australia; 4 School of Health Sciences, Faculty of Health, University of Newcastle, Callaghan, NSW, Australia; 5 School of Medicine and Public Health, University of Newcastle, Callaghan, NSW, Australia; 6 Interdisciplinary Educational Research Institute and Faculty of Education, University of Wollongong, New South Wales, Australia

## Abstract

**Background:**

The 'Healthy Dads, Healthy Kids' program was designed to help overweight fathers lose weight and positively influence the health behaviors of their children. The aim of the current study was to evaluate the previously established program in a community setting, in a large effectiveness trial.

**Methods/Design:**

The Healthy Dads, Healthy Kids community trial consists of three stages: (i) *Stage 1 - program refinement and resource development *(ii) *Stage 2 - community randomized controlled trial *(iii) *Stage 3 - community effectiveness trial*. The program will be evaluated in five Local Government Areas in the Hunter Valley Region of NSW, Australia. For the community randomized controlled trial, 50 overweight/obese men (aged 18-65 years) from one Local Government Area with a child aged between 5-12 years of age will be recruited. Families will be randomized to either the program or a 6-month wait-list control group. Fathers and their children will be assessed at baseline, post-intervention (3-months) and 6-months. Inclusion criteria are: body mass index 25-40 kg/m^2^; no participation in other weight loss programs during the study; pass a health-screening questionnaire; and access to a computer with Internet facilities. In the community trial, the program will be evaluated using a non-randomized, prospective design in five Local Government Areas. The exclusion criteria is body mass index < 25 kg/m^2 ^or lack of doctor's approval. Measures will be collected at baseline, 3-, 6- and 12-months. The program involves fathers attending seven face-to-face group sessions (three with children) over 3-months. *Measures*: The primary outcome is fathers' weight. Secondary outcomes for both fathers and children include: waist circumference, blood pressure, resting heart rate, physical activity, sedentary behaviors and dietary intake. Father-only measures include portion size, alcohol consumption, parenting for physical activity and nutrition and parental engagement. Process evaluation will determine the fidelity, dose (delivered and received), reach, recruitment and context of the program.

**Discussion:**

As a unique approach to reducing obesity prevalence in men and improving lifestyle behaviours in children, our findings will provide important evidence relating to the translation of Healthy Dads, Healthy Kids, which will enable it to be delivered on a larger scale.

**Trial registration:**

Australia and New Zealand Clinical Trials Register (ANZCTR): ACTRN12610000608066

## Background

Obesity is a serious public health concern associated with numerous negative health consequences [1]. Its prevalence is high and increasing [2], especially among men [3]. This is concerning given that men are less likely to self-identify as being overweight [4], attempt weight loss, or enroll in weight loss programs compared to women [5,6]. Although this is a concern, perhaps of greater worry is the impact obese fathers may have on their children. A recent study demonstrated that a father's parenting style was more influential than a mother's parenting style on their pre-school child's weight status [7]. Fathers' dietary intake has also been shown to be associated with their children's dietary intake [8]. Notably, a longitudinal study of more than 3200 families identified that having an overweight or obese father, but a healthy weight mother, increased the odds of a child becoming obese [9]. However, the reverse scenario (having an overweight mother with a healthy weight father) was not a significant predictor of childhood obesity. These findings provide emerging evidence that fathers are a key influence in shaping the home environment that may influence children's dietary and physical activity behaviors.

As parent behaviors, attitudes, parenting styles and practices have a profound influence on children's health behaviors [10,11], there is an urgent need to explore the feasibility and efficacy of lifestyle interventions that target parents and aim to influence the health behaviors of both parents and children. Despite the widely accepted notion that physical activity and dietary interventions for children should involve parents, the most appropriate strategies to recruit, engage and improve parental lifestyle behaviors have not been established [12,13]. Further, family-based lifestyle interventions have mainly engaged mothers [12]. We conducted the only experimental study targeting fathers, the *Healthy Dads, Healthy Kids *(HDHK) pilot, which examined the impact of a lifestyle program targeting fathers on children's physical activity and dietary habits [14].

The HDHK program was designed to help overweight fathers lose weight and role model positive health behaviors in order to positively influence the physical activity and dietary behaviors of their children. Preliminary studies have shown the program to be highly feasible and efficacious [14], with approximately 85% of fathers achieving a clinically important [15] weight loss (≥ 5% of their starting weight). In addition, children in the HDHK group significantly improved their physical activity levels and decreased their kilojoule intake, relative to those in the control group. The process evaluation also highlighted the feasibility of this approach with high levels of recruitment, retention, attendance and satisfaction of participants [14].

Despite the results of the HDHK pilot study, efficacy was established in a university-based research project that was delivered by highly qualified staff in a closely monitored trial. While efficacy is an essential first step and evaluates outcomes under ideal conditions, effectiveness measures the impact of an intervention when implemented in a real-world setting and represents a more realistic evaluation of the likely intervention effect [16]. While efficacy is more associated with internal validity, effectiveness relates more to external validity and examines whether an intervention can be translated to a real-world setting [16].

Therefore, the generalizability and effectiveness of the HDHK program requires testing in an appropriate trial to determine the impact of the program when delivered by trained community-based facilitators and with longer-term follow-up. This type of translational research will maximize the public health impact and reach of the HDHK program. There is an urgent need to translate obesity prevention and treatment programs with demonstrated efficacy into real-world settings [17] to reduce the significant void between research and practice and between the efforts of policy makers, researchers and practitioners in this field [18]. The aim of the current study is to implement and evaluate the HDHK program in an effectiveness trial set in the community.

## Methods/Design

### Study Design

The HDHK community trial consists of 3 major stages:

Stage 1 - Program refinement and resource development

Stage 2 - Community randomized controlled trial (RCT)

Stage 3 - Community effectiveness trial

The HDHK community program will be evaluated in five Local Government Areas (LGAs; Singleton, Maitland, Scone, Muswellbrook, Cessnock) in the Hunter Valley Region of New South Wales, Australia. In these areas, there are high levels of employment within the mining and associated manufacturing industries. In Australia, 52% of men who work in the mining industry are engaged in shift work [19], which is associated with increased health issues compared with working daytime hours and is an independent predictor of increased body mass index [20].

Stage 1 - Program refinement and resource development (2010)

One local government area (Singleton) was targeted in order to develop and test strategies for engaging communities, promoting the program among residents, and recruiting participants. Stage 1 was also used to further refine and develop key resources for families and for facilitators. For example, the session content was extensively reviewed and updated and more participant-centered learning experiences were embedded throughout the program. These developments were informed by the process evaluation of the pilot study [21] and from participant and facilitator feedback after running the HDHK program with a small group of fathers (n = 6) and their children from a primary school in Singleton in 2010. This trial of the HDHK program was delivered by PJM and the sessions were filmed to (a) assist with development of the training manual, and (b) be used as a facilitator training resource to demonstrate interactions with fathers that occur when implementing the program and visual demonstrations of the practical activities used in the interactive nights for fathers and their children. Details of the resources developed for the HDHK program are outlined in Table 1.

**Table 1:** HDHK program resources

Resource	Brief description	Included information
Facilitator manual	Comprehensive train-the-trainer manual detailing all information required for the delivery of the program	* Introduction * Successful facilitating * Background to the research * Session guides

Manual for Dads	Handbook for fathers that includes a summary of the information from the face-to-face sessions and additional background information	* Session 1: Weight loss fundamentals for men * Session 2: Raising children in an inactive world * Session 3: Ready to rumble with dad * Session 4: Healthy eating for families - dads matter * Session 5: Fun, fitness and fundamental movement skills * Session 6: Sustaining healthy lifestyles * Session 7: Weight loss is a journey not a destination

Dad's log book	Handbook that dads use as a working document	* Program activities: Energy calculations; sedentary behaviors; goal setting; eating habits * Homework activities: Children's screen time; family fitness activity; family cooking; children's food intake; cue words; family activity * Monitoring procedures: Pedometer chart; weight loss chart; goal recording

Website user guide	Handbook to guide fathers through the setup and use of the dietary and physical activity monitoring website	* Setting up a profile * Food and exercise diary * Check in diary * Charts and reports

Kid's Handbook	Includes tasks for children to complete each week with their dads that must be signed off with their dads (space to receive a sticker from facilitators if completed)	* Kids homework activities * Recipes to cook with dad

Green slips	Homework activity slips given to fathers at the end of each session	* Activities are designed to align with the principles presented at the intervention session and any activities detailed in the Dad's log book and Kid's handbook (e.g. cooking meals with kids, family fundamental movement skills circuit, rough and tumble games with dad)

Stage 2 - Community RCT design

With the facilitator training and resources developed, the impact of the program in a community setting will be evaluated with trained local facilitators, employing a RCT design.

#### Participants

Overweight or obese (BMI between 25 and 40 kg/m^2^) fathers (aged 18-65 years) with a child aged between 5 and 12 years of age (i.e. primary school age) will be recruited from the local communities using the following strategies: local media releases (print, TV and radio), school newsletters items, school-based presentations, fliers distributed through community notice boards and local businesses, paid advertisements in local newspapers, local networks of sports clubs, service clubs, local government, and community health facilities. Fathers will be screened for eligibility via telephone. Ethics approval has been obtained from the University of Newcastle Human Research Ethics Committee. Written informed consent will be obtained from the fathers prior to their participation as well as child assent.

Fifty fathers will be recruited from one LGA (Maitland) and randomized to either the HDHK program or a 6-month wait-list control group. Fathers (and their children) will be stratified by BMI category (overweight [25-29.9 kg/m^2^], obese class 1 [30-34.9 kg/m^2^], obese class 2 [35-40 kg/m^2^]) and randomized using a computer-based random number-producing algorithm, to intervention or control group. To ensure concealment, the sequence will be generated by a statistician and given to the project manager. Randomization will be completed by a research assistant not involved in assessments and the allocation sequence will be concealed when enrolling participants. Fathers and their children will be assessed at baseline, post-intervention (3-months) and 6-months (3-months post-intervention).

#### Inclusion/exclusion criteria

Ineligibility criterions consist of a history of major medical problems such as heart disease in the last five years, Type 1 diabetes, orthopedic or joint problems that would be a barrier to physical activity, recent weight loss of ≥ 4.5 kg and taking medications that might affect body weight. All fathers need to have a BMI between 25 and 40, have internet access and will be asked to not participate in other weight loss programs during the study. Fathers will complete a pre-exercise risk assessment screening questionnaire [22]. Any participant over the age of 40 will be required to provide a doctor's clearance before enrolling in the study.

#### Sample size

For the RCT, based on 80% power to detect a significant weight loss difference between groups of 3 kg, assuming SD = 5 (*P *= 0.05, two-sided) a sample size of 20 fathers for each group will be needed at 6 months. Assuming a 20% attrition rate [14], a total sample of 50 fathers will be required. Participants will be blind to group allocation at baseline assessment. Once baseline assessments are completed, participants will receive a sealed envelope with a note advising their group allocation. The wait-list control group will receive no information or intervention before attending the 3- and 6-month follow-up assessment sessions. There will be a 6-month wait-list control group as (a) ethically every overweight man is deserving of an intervention and is unlikely to remain weight stable; (b) randomization to 'no' intervention for a greater length of time is unlikely to be acceptable and is likely to lead to high attrition compromising study integrity.

Stage 3 - Community effectiveness trial design

When translating evidence-based programs into real-world settings, ongoing modifications are required based on the unique characteristics of the settings and populations. Modifications will be made to the program based on both outcome and process evaluation from the community RCT and each subsequent program implementation in 2011 and these will be documented. The goal will be to develop a comprehensive model of a real-world intervention for application in communities through updating materials, developing standardized train-the-trainer education and resources and ongoing evaluation of the implementation of the intervention with a final evaluation conducted in 2012.

The HDHK program will be evaluated for effectiveness in the community roll-out using a non-randomized, prospective design. This evaluation will be conducted in both 2011 and 2012 in the five local government areas. The exclusion criteria is minimal to align with a true translational model and will only include BMI < 25 or lack of doctor's approval. Measures (detailed below) will be collected at baseline, post-intervention, and 6 and 12 months post-baseline. We aim to recruit 15-20 fathers from each area. Results from each program will be pooled and it is expected at least 200 families will participate in the community trial of the HDHK program.

### The HDHK Intervention

The aims of the program are to help fathers achieve their weight loss goals, become healthy role models, and promote healthy behaviors for their children. Table 2 details the specific HDHK program content for each session. The 3-month HDHK program involves fathers attending seven face-to-face group sessions (90 minutes each). Four group sessions are for fathers only, and three sessions involve both the fathers and their children. These practical sessions will be conducted in local school halls and delivered by two trained facilitators.

**Table 2:** HDHK sessions and program content

Session focus	Session detail
1. 'Weight loss for men' (Dads)	* Program rationale * Importance of fathers and their influence on children * Energy balance and weight loss * Nine key weight loss tips for men * Website use for eating and activity diaries

2. 'Raising active children in an inactive world' (Dads)	* Obesogenic environments * Physical activity levels, trends and benefits * Physical activity recommendations * Physical activity goals for Dads * Ideas for fitness/activity at home

3. 'Ready to rumble with Dad' (Dads & Kids)	* Rough and Tumble Play * Fun Fitness circuits * Fun and active games

4. 'Healthy eating for families - Dads matter' (Dads)	* Healthy eating benefits * Child nutrition * Food based guidelines * Role of fathers in healthy home eating environments * Authoritative feeding practices * Reading food labels

5. 'Fitness, fun and fundamental movement skills (FMS)' (Dads & Kids)	* FMS skills circuit * Rough and Tumble activities * Partner fitness challenges

6. 'Sustaining healthy lifestyles' (Dads)	* Planning meals * Australian Guide to Healthy Eating * Recommended daily intakes * Why we eat food? * Support and strategies for successful dietary changes and relapse prevention

7. 'Weight control is a journey, not a destination' (Dads & Kids)	* Program revision * Challenges and solutions * Rough and tumble activities * Ball and game skills

The HDHK program is based on Social Cognitive Theory (SCT) [23] and Family Systems Theory (FST) [24]. The following SCT constructs are targeted and operationalized: self-efficacy, goals/intention, outcome expectations, perceived facilitators and barriers to changes, and social support. FST postulates a complex theoretical framework of reciprocal relationships among family members. That is, when a parent changes his or her physical activity and dietary behaviors this will be reflected in the child's behavior [25]. HDHK provides fathers with the knowledge and skills important for long-term behavior change. HDHK teaches fathers about the importance of spending quality time with their children and uses healthy eating and physical activity as the medium to engage fathers with their children. By including the children in the HDHK sessions, children's enthusiasm for father-child activity can reinforce the shift in family lifestyle.

Sessions on healthy eating for families focus on various aspects of parental influence on children's dietary intake incorporating Satter's 'trust' paradigm [26], which suggests parents should supply healthy foods and a supportive eating environment and children can decide when and how much to eat. Sessions focus on promoting a 'do as I do' and not a 'do as I say' philosophy and making small changes, building on initial success and setting up a home environment where sustainable healthy family eating patterns can be established. The physical activity and dietary component focus on an authoritative parenting style to facilitate better dietary and activity choices for children and have been informed by the dietary intervention previously implemented in the successful HIKCUPS child obesity intervention [27,28]. Although mothers do not attend sessions, fathers are encouraged to enhance social support for their child's efforts and consider strategies to involve mothers.

### Facilitator training

The HDHK program targets male facilitators with relevant discipline knowledge and experience. Although experienced physical education (PE) teachers are not specifically targeted, they are ideally suited to deliver the program. PE teachers have experience in teaching children, qualifications in education, expertise in health-related areas, and access to and understanding of schools. Importantly, they have the distinct skills and expertise to safely and effectively run the practical sessions. The training workshops are 6-8 hours of face-to-face contact and are designed to cover the background information to the program, session-by-session content knowledge, and the mechanics of the program (leading group sessions, role play, using the facilitator manual, homework activities, responses to common questions/problems and evaluation procedures).

A network of trained facilitators will be established to deliver the program in their local areas. Two facilitators will be paid to deliver the program. The lead facilitator's role is to present all materials and lead the delivery of the sessions. The co-facilitator provides a supporting role for the lead during all sessions (including equipment management, assisting in concept explanations and management of group-based activities), administrative support prior to (participant weigh-in, attendance sheets and homework compliance) and post (participant feedback questionnaires) all sessions. Both facilitators are responsible for completing the facilitator session feedback (independently) and for liaising with each other regarding equipment and resource requirements for each session.

### Measures

A variety of data collection methods will be used to assess program impact, implementation, participation and fidelity. For the community RCT, participants will be blinded to group allocation at baseline assessment. Baseline assessments will be made 1-2 weeks before program commencement. Assessors will be trained by the same experienced researcher, and for anthropometric measurements, the protocols used are those prescribed by the International Society for the Advancement of Kinanthropometry [29]. A summary and brief description of the measures used for both the RCT and community trial programs is described in Table 3 with more specific details provided below.

**Table 3:** Overview of HDHK RCT and community trial measures

Measure	RCT	Community trial
PHYSICAL MEASURES		
Weight	**Father & child(ren)**: Digital scale	**Father & child(ren)**: Digital scale
Height	**Father & child(ren)**: Portable stadiometer	**Father & child(ren)**: Portable stadiometer
BMI	**Father: **(kg/m^2^). **Child(ren): **(kg/m^2^) and z-scores.	**Father: **(kg/m^2^). **Child(ren): **(kg/m^2^) and z-scores.
Waist circumference	**Father**: 1) level with the umbilicus and 2) the widest point. **Child(ren)**: 1) level with the umbilicus and 2) the narrowest point + z-scores.	**Father**: 1) level with the umbilicus and 2) the greatest circumference point. **Child(ren)**: 1) level with the umbilicus and 2) the narrowest point + z-scores.
Blood Pressure and Resting Heart Rate	**Father & child(ren)**: Digital electronic monitor.	Not measured

PHYSICAL ACTIVITY AND SEDENTARY BEHAVIOR		
Physical activity (PA)	**Father & child(ren)**: Yamax200 pedometers for 7 consecutive days.	**Father**: Modified version of the Godin Leisure-Time Exercise Questionnaire (GLTEQ) [39]. **Eldest child **(Father proxy): Children's Leisure Activities Study Survey (CLASS) questionnaire [41].
Sedentary Behaviors	**Father**: Modified Sitting Questionnaire [42,43]. **Eldest child**: (Mother proxy) modified (CLASS) [41]	**Father**: Modified Sitting Questionnaire [42,43]. **Eldest child **(Father proxy): Indoor based activities section of CLASS [41].

NUTRITION AND DIETARY BEHAVIORS		
Dietary intake	**Father**: Australian Eating Survey (AES) [44]. **Eldest child **(Mother proxy): Australian Child and Adolescent Eating Survey (ACAES) [46].	**Father**: Modified AES relating to 8 key nutritional messages **Eldest child **(Father proxy): Modified AES relating to 8 key nutritional messages.
Portion size	Not measured.	**Father: **Photographs from the Dietary Questionnaire for Epidemiological Studies Version 2 [49].
Alcohol Consumption	Not measured	**Father**: Modified Alcohol Use Disorders Identification Test 2009 [52].
Parenting strategies for eating & PA	**Father and mother **(about eldest child): Parenting Strategies for Eating and Activity Scale (PEAS) [56].	**Father **(about eldest child): PEAS [56].
Child Feeding Habits	**Mother **(about eldest child): Child Feeding Questionnaire [55].	Not measured

PARENTING STRATEGIES		
Parenting for PA & nutrition	**Father **(about eldest child): A combination of parenting measures from previously validated sources with some new items	**Father **(about eldest child): A combination of parenting measures from previously validated sources with some new items
Parental engagement	**Father **(about eldest child): Parental Engagement Questionnaire [63,64] and additional questions on father's physical play	**Father **(about eldest child): Modified Parental Engagement Questionnaire (also questions relating to the father's perceptions of father's physical play with their eldest child).
Attitudes to rough and tumble play	Not measured.	**Father**: Semi structured interview

ADDITIONAL INFORMATION		
Socio-demographic information	**Father & child(ren)**: Age and socioeconomic status	**Father & child(ren)**: Age and socioeconomic status

#### Demographic characteristics

Background details and socio-demographic variables will be collected by questionnaire including age, marital status, occupation, gross annual family income, educational level, ethnic origin, language spoke at home, socioeconomic status (SES) and postcode. SES is based on postal code of residence using the Index of Relative Socioeconomic Advantage and Disadvantage from the Australian Bureau of Statistics census-based Socio-Economic Indexes for Areas (SEIFA) [30].

#### Primary outcome measure

The primary outcome measure is body weight of the fathers at 6-month follow-up. Weight will be measured with fathers wearing light clothing, without shoes on a digital scale to 0.1 kg (model CH-150 kp, A&D Mercury Pty Ltd, Australia). Weight will be recorded twice and the average of the two measures reported. The same protocol will be followed to record children's weight.

#### Secondary outcomes

##### BMI

will be calculated using the standard equation (weight [kg]/height[m]^2^). Height will be measured to 0.3 cm using the stretch stature method and a stadiometer (VR High Speed Counter) (Harpenden/Holtain, Mentone Education Centre, Morrabin, Victoria). Height will be recorded twice and the average of the two measures reported. For children, height and weight will be used to calculate BMI (kg/m^2^) and age- and sex-adjusted standardized scores (*z*-scores) based upon the UK reference data [31] and LMS methods [32] will be used. International Obesity Task Force cut points will be used to determine overweight or obesity [33].

##### Waist circumference

Waist circumference will be measured at two points for all participants. In fathers: (i) level with the umbilicus and (ii) at the greatest circumference between the lower costal border and the iliac crest; and in children: (i) level with the umbilicus and (ii) at the narrowest point. Each measurement will be recorded with a non-extensible steel tape (KDSF10-02, KDS Corporation, Osaka, Japan). Two measures will be taken and if the measures differ by more than one centimeter, a third will be recorded. The average of the measures will be reported and a waist *z*-score calculated for children [34].

##### Blood Pressure and Resting Heart Rate (RHR)

Systolic and diastolic blood pressures will be measured using a NISSEI/DS-105E digital electronic blood pressure monitor (Nihon Seimitsu Sokki Co. Ltd., Gunma, Japan) under standardized procedures. Participants will be seated for at least five minutes before blood pressure and RHR is recorded. Blood pressure and RHR will be measured three times and the average of the three measures will be reported.

##### Physical activity (objective measure)

Yamax SW700 pedometers (Yamax Corporation, Kumamoto City, Japan) will be used to objectively measure physical activity; they are considered to be more reliable than other brands of pedometers [35]. They have been validated in children [36] and adults [37]. Participants will be asked to wear pedometers for seven consecutive days and maintain their normal routine. At baseline assessments, participants will be instructed on how to attach the pedometers (at the waist on the right hand side) and asked to remove the pedometers only when sleeping, when the pedometer might get wet (e.g. swimming, showering) or during contact sports. At the end of the day, participants are instructed to record their steps and reset their pedometers to zero. Once seven days of monitoring has been completed, participants are instructed to place the pedometer and record sheet in the prepaid envelope provided and return to the research team. Participants will be included in all analyses if they have completed at least four weekdays of pedometer monitoring and one weekend day.

##### Physical activity (Father - questionnaire measure)

Self-reported physical activity will be collected using a modified version [38] of the validated Godin Leisure Time Exercise Questionnaire (GLTEQ) [39], that asked participants to report the average number of times per week, in the past month, they engaged in vigorous (rapid heartbeats, sweating), moderate (not exhausting, light perspiration) and mild (minimal effort and no perspiration) intensity physical activity, for a minimum of 10 minutes per session. As specified by the GLTEQ, weights of 9, 5 and 3 will be applied to vigorous, moderate and mild frequencies respectively and a summary score of the weighted physical activity per week will then be computed by adding vigorous, moderate and mild activity frequencies [39] to obtain an overall GLTEQ score. Participation responses for the moderate and vigorous activity categories will also be added to obtain total minutes of moderate and vigorous minutes per week. Physical activity measures will be converted into metabolic equivalent (MET) scores (i.e., MET-minutes/week) by multiplying reported weekly minutes in each intensity category by each respective MET-minute value (i.e., moderate intensity minutes multiplied by 4 MET and vigorous intensity by 7.5 MET) and mild intensity by 2.5 [40]. [For example, one hour (60 min) of moderate-intensity exercise would be equal to 240 MET-min of activity (60 minutes times 4 METs)].

##### Physical activity (Eldest child - questionnaire measure)

Assessed in the RCT by the mother using a modified Children's Leisure Activities Study Survey (CLASS), which has been validated in children 5 - 6 and 10 - 12 year of age [41]. In the community trial, fathers will complete a version of CLASS to obtain a measure of physical activity time for their children.

##### Sedentary behavior

For the eldest child in the RCT, the mother will complete a modified version of the CLASS, which has been validated in children 5 - 6 and 10 - 12 year of age [41]. In the community trial, fathers will complete a version of the CLASS to obtain a measure of time spent in various sedentary behaviors. Only questions relating to indoor leisure activities will be used (small screen recreation, homework, talking, reading, music and craft activities). To assess their sitting time, fathers will complete an adaptation of the Sitting Questionnaire, which has been shown to be both a valid and reliable measure of sitting time in various domains [42,43].

##### Dietary intake (Fathers)

will be assessed in the RCT using the Australian Eating Survey (AES). AES is a 120-item semi-quantitative Food Frequency Questionnaire (FFQ), used previously in Australian youth up to 16 years [44] and currently being validated in both adult males and females. Portion sizes for individual food items were generated by the Australian Bureau of Statistics (ABS) [45] and unpublished data from the 1995 Australian National Nutrition Survey; or the "natural" serving size for common items such as a slice of bread. Subjects are asked about frequency of their consumption over the previous six months with frequency options ranging from 'Never' up to '4 or more times per day' but varying depending on the food item. Twenty-one questions relate directly to the intake of vegetables and 11 questions relate to fruit. Seasonal availability of some fruits will be considered in the nutrient analysis. Nutrient intakes from the AES will be computed from the most current food composition database of Australian foods available, the Australian AusNut 1999 database (All Foods) Revision 17 and AusFoods (Brands) Revision 5 (Australian Government Publishing Service, Canberra) to generate individual mean daily macro-and micro-nutrient intakes. The AES includes questions about the total number of daily serves of fruit, vegetables, bread, dairy products, eggs, fat spreads, sweetened beverages and snack foods, as well as asking the type of bread, dairy products and fat spreads used. Twelve questions relate to food-related behaviors, including items on frequency of take-away food consumption and eating while watching television.

Dietary intake for fathers in the community trials will be assessed using a modified version of the AES. Eight questions related to the key nutritional messages of the program will be drawn from the full AES survey (milk type, fruit intake, vegetable intake, takeaway food intake, meals whilst watching television, sweetened drink intake, fruit juice intake, and snack frequency).

##### Dietary intake (Eldest child)

For the eldest participating child in the RCT, their mothers will complete the Australian Child and Adolescent Eating Survey (ACAES), developed and validated for use with Australian children, aged 10 to 16 years [46] and previously shown to be sensitive to changes up to two years [47]. ACAES has also been validated for younger children aged 5-9 years for parent-reported fruit and vegetable intake using plasma carotenoid concentrations [48]. Children's dietary intake will be adjusted relative to body weight and reported as kJ/kg. At follow-up assessments, participants and mothers will be instructed to report on the previous 3-month dietary intake. Dietary intake for the eldest child in community trials will be assessed using a modified version of the ACAES, as described previously, and completed by the fathers.

##### Portion size

will be assessed using portion size photographs from the Dietary Questionnaire for Epidemiological Studies Version 2 (DQES v2), FFQ from the Cancer Council Victoria [49]. These photos are used to calculate a single portion size factor (PSF) to indicate whether on average a person eats median size serves (PSF = 1), more than the median (PSF > 1), or less than the median (PSF < 1) serve sizes for main meals. The DQES was developed specifically for use in Australian adults by the Cancer Council of Victoria as an update of a FFQ used in a cohort of Australian volunteers aged 40-69 years. Both the development of the questionnaire [50] and its validation have been reported previously [51].

##### Alcohol consumption (for fathers)

Alcohol consumption will be measured using an adaptation of the Australian Government Department of Veteran Affairs, Alcohol Use Disorders Identification Test (AUDIT) 2009 [52]. This instrument has been shown to be a valid and reliable measurement tool in determining alcohol use disorders and alcohol misuse [53,54].

##### Child feeding habits

The Child Feeding Questionnaire (CFQ) is a 31-item validated questionnaire, designed for completion by parents of children aged 2 to 11 years [55]. The CFQ measures parental beliefs and attitudes regarding child-feeding practices across seven domains. Perceived parent overweight is a 4-item scale that measures parent's perception of their own weight at various life stages including childhood, adolescence, early adulthood, and currently. Perceived child overweight is a 6-item scale that measures the parent's perception of their child's weight at various life stages including the first year of life, as a toddler through to child's weight at 6th to 8th grade at school. As these are age-based questions, perceived child weight factor will be determined by the answers to the two age appropriate items only (questions 8-10). Perceived feeding responsibility is a 3-item scale measuring how responsible a parent feels for feeding their child. Concern about child overweight is a 3-item sub-scale that reflects the degree to which the parent is concerned their child is or will become overweight. Restriction is an 8-item scale that measures the parents' attempts to control their child's eating by restricting access to likable foods. It concerns the restriction of both the type and amount of food. Pressure to eat is a 4-item scale that measures the degree to which a parent encourages the child to eat by behaviors, such as insisting that the child eat everything on their plate. Monitoring is a 3-item scale that examines the degree to which a parent keeps track of their child's consumption of energy dense snack foods. Mean scores for each of the seven feeding domains will be calculated for all children at each time period.

##### Parenting strategies for eating and activity

will be assessed using the Parenting Strategies for Eating and Activity Scale (PEAS), which has been shown to be a valid measure of parenting strategies related to children's obesity-related behaviors [56]. During the RCT, both father and mother of the eldest child will complete this measure for the investigation of parenting strategy differences/similarities within family groups for the same child. For the community trial, only fathers will complete this measure.

##### Parenting for Physical Activity and Nutrition

This questionnaire uses a combination of previously validated parenting measures focusing on: (i) Parenting for physical activity (fathers logistic support for PA [57], parental role modeling [57,58], parental beliefs [59], and co-physical activity [59]); (ii) Parenting for nutrition (shared meal time frequency [60], parental role modeling [57,58], parental policies supporting healthy eating [58], and parental beliefs [59]); and (iii) Parenting for combined physical activity and nutrition (parental confidence for effective child PA and nutrition [61] and parental intention to encourage healthy eating and PA [62]).

##### Parental engagement

will be assessed in the RCT by fathers completing a Parental Engagement Questionnaire which includes questions from the Child Rearing Questionnaire [63] and the National Longitudinal Survey of Children and Youth [64] to assess parental warmth, hostility and control. These questions have been utilized in the Longitudinal Study of Australian Children [65]. Additional questions on father's physical play were designed for this study. For the community trial, fathers will complete a modified Parental Engagement Questionnaire, with additional questions directed at the father's perceptions of father's physical play (rough and tumble) [66] with their eldest child.

##### Attitudes to rough and tumble play

will be assessed using a semi-structured interview. The fathers' responses to the interview questions will identify the parameters of rough and tumble play and other types of physical play and lead to an operational definition of rough and tumble play that can then be used in future research.

### Process evaluation

As lack of monitoring of program integrity and the intervention dose received by participants have been identified as methodological limitations of obesity prevention programs [67], we will explore the consistency with which the HDHK program is delivered in the RCT and community trials. This will help to determine the quantity of the intervention to which the families are exposed. The comprehensive and systematic process evaluation framework outlined by Saunders et al [68] and initially proposed by Steckler and Linnan [69] has been adapted for the HDHK community process evaluation. These major elements include fidelity, dose (delivered and received), reach, recruitment and context (Table 4) and include both formative and summative processes.

**Table 4:** HDHK process evaluation procedures

	*Process Evaluation Question*	*Data Sources*	*Tools/Procedures*	*Timing of Data Collection*	*Data Analysis or Synthesis*	*Reporting*
*Fidelity*	To what extent was the intervention implemented consistently with the underlying theory and philosophy? ^#^	Facilitators HDHK research staff	* Independent self-reported checklist from Lead and Co Facilitator regarding the amount of pre-prepared content delivered and any non-delivered content * Observation checklist of delivered content from independent observer to validate facilitator self-report checklist	* At the conclusion of each session (Checklists will be faxed to research staff post-session) * At least two observations per seven session program	* Calculated score based on percentage of intended content included * Percentage difference score of researcher versus facilitator average of intended content included	* Formative - informal feedback to staff prior to the next intervention session * Summative - reported after the program is complete

*Dose delivered*	To what extent were all intervention components implemented?	Facilitators HDHK research staff	* Independent self-reported checklist from Lead and Co Facilitator regarding the amount of pre-prepared content delivered and any non-delivered content (theoretical and practical) * Observation checklist of delivered content from independent observer to validate facilitator self-report checklist	* At the conclusion of each session (Checklists will be faxed to research staff post-delivery) * At least two observations per seven session program	* Calculated score based on percentage of intended content included * Percentage difference score of researcher versus facilitator average of intended content included	* Formative - informal feedback to staff prior to the next intervention session * Summative - reported after the program is complete

*Dose received*	How did participants react to the activities presented during the intervention?	Participants	* Participant evaluation of each of the attended sessions (enjoyment, amount learnt, ease of understanding content, relevance to self, likelihood of strategy use, likes and suggested changes) * Post-program evaluation (likes, dislikes, comments, attitudes before, attitudes after, program activities, facilitators, resources)	* Post-session * Post-program	* Quantitative analysis of session scores; Qualitative analysis of likes and suggestion themes per session and across the program	* Summative - reported after the program is complete

	To what extent did participants engage in recommended follow-up behavior?	Participants	* Father's log book and green slip homework activities; Kid's log book	* Father's log book (post-program); Kids log book (during and post program); Father's green slip homework (weekly)	* Level of follow-up activity completed included as a factor during quantitative analysis	* Summative - reported after the program is complete

*Reach*	What proportion of fathers attended greater than 70% of sessions (5 of 7)?	Facilitators	* Session roll for attendance	* Recorded at each session	* Level of attendance included as a mediator during quantitative analysis	* Summative - reported after the program is complete

*Recruitment*	What recruitment activities were used to attract organizations and participants? What recruitment activities attracted participants to the program?	HDHK research staff HDHK research staff	* Marketing spread sheet maintained by research staff (target market, activity, date, response); Recruitment/screening spread sheet recording the date of first contact by a potential participant * Identification of the most influential marketing method by the potential participant and any other marketing they had been exposed to	* Each time recruitment activities were undertaken * Recorded at initial phone screening of potential participant	* Narrative description of procedures; graphing of marketing methods and participant response dates to identify successful trends * Narrative description of influential recruitment strategies; Calculate percentages of most influential strategies leading to participant contact with the research team	* Formative - reported weekly to identify responses to marketing methods used; Summative - reported after the program is complete to inform next cohort * Formative - reported weekly to identify responses to marketing methods used; Summative - reported after the program is complete to inform next cohort

*Context*	What barriers to participation were experienced by the participants?	Facilitators using participant information	* Focus groups as part of the program to identify barriers to implementing the information/activities covered during the program	* Session 6 of 7 (facilitators to record the barriers identified by participants)	* Themes identified through qualitative analysis	* Summative - reported after the program is complete

	What aspects of the program did and didn't appeal to the participants?	Participants	* Post-program evaluation (likes, dislikes, comments, attitudes before, attitudes after, program activities, facilitators, resources)	* Post-program	* Themes identified through qualitative analysis and statistical analysis of questionnaire	* Summative - reported after the program is complete

^# ^Underlying theory and philosophy is embedded in the content given to facilitators to deliver the participants. If all content is delivered as per the program package, consistence of theory and philosophy is said to have been achieved.

### Community Program Feasibility

The feasibility of the program as a community intervention will also be evaluated using a number of metrics including recruitment (achievement of target sample size), attendance (at program sessions), retention (retention rates at 6-month follow-up) and participant perceptions collected from fathers about program satisfaction via a questionnaire (end of program evaluation) that focuses on: open ended feedback of positives and negatives of the program; multiple choice questions regarding prior weight-loss experiences and reasons for joining the program; and Likert scale (5-point) questions anchored from (1) strongly disagree to (5) strongly agree regarding perceptions of program content, program structure, quality of facilitators, quality of program resources, and perceived changes made during the program. Additional information regarding the long term feasibility of the program will be obtained using a semi-structured post program phone interview and a long term process evaluation questionnaire (12 month post program evaluation).

### Sustainability planning

The project is based on the principles of community consultation and will actively seek the ideas and opinions of the communities in which the project will run, including via community forums and leadership from a community advisory panel. Community advisory panel members will represent a broad range of areas including education, local government, health, sports groups and service clubs. The communities will be consulted on the best approaches to engage and recruit participants and run the program in a sustainable manner. The community consultations will also help to identify key local individuals and organizations that can potentially assist in embedding the HDHK program in the local community. The consultations will also assist in identifying suitable local venues for the HDHK program and gaining information about local conditions which may impact on the delivery of the HDHK program (e.g. timing of delivery). Additionally, in order to draw on the strengths and resources of each community and to promote capacity building for sustainability of the program, appropriately qualified community members will be invited to train as facilitators who will conduct the program in their communities. Community consultation meetings will be held in each LGA prior to commencing the program. This will facilitate collaborative partnerships between the researchers and community, and contribute to the sustainability of the program within each community. Any interested community member will be able to attend.

Stage 1 focuses on developing training and implementation manuals that will provide step-by-step instructions on how to run the program. These resources will allow the community to continue to run the program indefinitely after the three year program has been completed. This, along with the training and implementation resources, will provide the basis for gaining support for investment in these strategies by other interested communities, and more importantly governments and health care policy makers. We have also developed a HDHK website http://www.healthydadshealthykids.com.au which provides information for potential participants and other interested parties.

### Data Analysis

Analyses will be performed using PASW Statistics 19 (SPSS Inc. Chicago, IL). Data will be presented as mean (SD) for continuous variables and counts (percentages) for categorical variables. Characteristics of completers versus dropouts will be tested using independent *t *tests for continuous variables and chi-squared (χ^2^) tests for categorical variables. The significance level will be set at 0.05 for all analyses. Analyses will be performed separately for fathers and children and included all randomized participants. Generalized linear mixed models (GLM) will be fitted with an unstructured covariance structure for all primary and secondary outcomes. Differences between means and 95% confidence intervals (CIs) will be determined using GLM.

GLM will be used to assess all outcomes for the impact of group (Intervention and control), time (treated as categorical with levels baseline, 3-months and 6-months) and the group-by-time interaction, these three terms forming the base model. Baseline scores for subjects who dropped out at 3-months and/or 6-months will be retained consistent with an intention-to-treat (ITT) analysis. Mixed models are more robust to the biases of missing data, and provide better control of Type 1 and Type 2 errors than last observation carried forward (LOCF) in Analysis of Variance [70]. Similarly, imputation methods such as LOCF or baseline carried forward may bias results towards the null in obesity trials where untreated overweight men are likely to increase their weight. Age, SES and sex of the child will be examined as covariates to determine if they contributed significantly to the models. If a covariate is significant, two-way interactions with time and treatment will also be examined and all significant terms will be added to the final model to adjust the results for these effects. Effect sizes will be determined using Cohen's *d *[71] and calculated using mean differences and the pooled standard deviation of the group (*d *= M_1 _- M_2_/σ_pooled_). We will also examine a range of secondary outcomes to support the primary outcome (e.g. reduction in waist circumference, increase in physical activity, reduction in kilojoule intake). Additional exploratory models will be fitted to examine if the fathers who have the greatest reduction in weight are also those who have the greatest improvements in the secondary outcomes.

For the community trial, the mean change between pre and post-intervention outcomes will be analyzed using paired Student's t-test when change data are normally distributed. Wilcoxon matched pairs signed rank tests will be used for non-parametric tests. Per protocol analyses for both the RCT and the community trial will be performed for those who attended at least 70% of HDHK sessions and the follow-up assessment visits.

## Discussion

The primary aim of this study is to evaluate the impact of the HDHK program in a community setting. This study will test the previously established efficacious HDHK program [14] in a larger effectiveness trial in five local government areas in the Hunter Valley in NSW, Australia. This is an area of NSW with higher than average obesity prevalence and a high population of shift workers, making this a high health risk population area [19]. Therefore, the examination of the effectiveness of a successful father-focused weight loss program within a community setting is of great importance.

Translating research results into evidence-based community programs that are widely disseminated is a major public health challenge [18]. The programs with the most potential to prevent unhealthy weight gain in children are those that are evidence-based, readily accessible, require minimal resources, and can be delivered in community settings. As a unique approach to reducing obesity prevalence in men and improving lifestyle behaviors in children, we plan to investigate the effectiveness of targeting overweight fathers to elicit changes in their own diet and physical activity habits and those of their children. Our findings will provide important information relating to the translation of HDHK which will enable it to be delivered on a larger scale and enable further studies to address long-term impact and ongoing evaluation. In addition, the HDHK community program will add to the health promotion services in regions typically having fewer allied health professionals compared to major cities.

Our community-based participatory research approach focuses on local relevance of health issues and collaborative partnerships between the researchers and community; builds on the strengths and resources of a community; and promotes capacity building. Translating efficacious interventions into community settings has the potential to make a significant public health impact. The potential public health benefits relate to our key research outcome, which is the improvement in the overall health and quality of life of participants (both fathers and children) through (a) positive changes in behaviors relating to physical activity and food intake (b) positive role modeling and engagement of fathers in their children's health and life and (c) community benefits through reductions in the burden of disease and direct/indirect health costs that are associated with poor lifestyle behaviors.

## Competing interests

The authors declare that they have no competing interests.

## Authors' contributions

The study chief investigators PJM, DRL, CEC, RC, RF, ADO, RCP, TB and VC were responsible for identifying the research question, design of the study, obtaining ethics approval, and the acquisition of funding and overseeing study implementation. Research assistants MDY, AM, and PhD student AL have contributed to the development of intervention materials, recruiting participants and study implementation. All authors were responsible for the drafting of this manuscript and have read and approved the final version.

## Pre-publication history

The pre-publication history for this paper can be accessed here:

http://www.biomedcentral.com/1471-2458/11/876/prepub
